# Supplementary material for: Lattice oxygen activation and local electric field enhancement by co-doping Fe and F in CoO nanoneedle arrays for industrial electrocatalytic water oxidation
Source: Nat Commun. 2024 Feb 3;15:1012. doi: 10.1038/s41467-024-45320-0 (PMC10837452; doi:10.1038/s41467-024-45320-0)
Supplement: Supplementary file 1 — Supplementary Information [file 41467_2024_45320_MOESM1_ESM.pdf]

Supplementary Information

Lattice oxygen activation and local electric field enhancement by co-doping Fe and F in CoO nanoneedle arrays for industrial electrocatalytic water oxidation

Pengcheng Ye[1,+], Keqing Fang[1,+], Haiyan Wang[1,*], Yahao Wang[1], Hao Huang[2,*], Chenbin Mo[1], Jiqiang Ning[3], and Yong Hu[4,*]

[1]Key Laboratory of the Ministry of Education for Advanced Catalysis Materials, Department of Chemistry, Zhejiang Normal University, Jinhua 321004, China.

[2]Department of Microsystems, University of South-Eastern Norway, Borre 3184, Norway.

[3]Department of Optical Science and Engineering, Fudan University, Shanghai 200438, China.

[4]College of Chemistry and Materials Engineering, Zhejiang A&F University, Hangzhou 311300, China.

[+]These authors contributed equally.

*e-mail: yonghu@zafu.edu.cn (Y. Hu)

chemwhy@zjnu.edu.cn (H. Wang);

huanghao881015@163.com (H. Huang)

This file includes:

Supplementary Methods

Supplementary Figures 1 to37

Supplementary Tables 1 to 8

Supplementary References

Table of Contents

1. **Supplementary Methods**........S3
2. **Supplementary Figures**........S8
3. **Supplementary Tables**........S45
4. **Supplementary References**........S53

1. Supplementary Methods

Theoretical simulations

To investigate the OER electrocatalytic mechanism of the doped CoO catalysts, density functional theory (DFT) was carried out using Materials Studio software. In this work, we chose the (200) plane as an active interface, and the corresponding slabs of Fe, F-CoO, F-CoO, Fe-CoO, and CoO were established based on 2 × 2 × 2 supercell of CoO crystal structure, respectively. A vacuum layer of 30 Å was incorporated into the slabs to avoid periodic interaction. The computations were conducted based on the DFT using Dmol3 and CASTEP code. Dmol3 code was utilized to optimize the structure of slabs and calculate the free energies, CASTEP code was employed for partial density of states (PDOS) of these optimal slabs. DFT calculations of these slabs were computed by using a generalized gradient approximation (GGA) of exchange-correlation functional in the Perdew, Burke, and Ernzerhof (PBE)[1]. The structure was fully optimized until the force on each atom is less than 10^{-3} Ha/Å. Moreover, to investigate the effect of the local electric field on the OER process, an electric field of 1.58×10^{-2} V/Å based on the finite element analysis numerical simulations, was introduced into the calculation of free energy on Fe, F-CoO slab. PDOS calculation was performed by GGA+U functional with additional Coulomb potential (U_{Fe} = 3.0 and U_{Co} = 3.1 eV) for 3d-orbit, a plane-wave energy cut off of 500 eV was used together with norm-conserving pseudopotentials, and the Brillouin zone was sampled with a 2 × 2 × 1 Monkhorst–Pack grid[2].

The free energy (ΔG) was computed from

$$\Delta G = \Delta E + ZPE - T\Delta S + ne\Delta U \quad (1)$$

where ΔE is the total energy, ZPE is the zero-point energy, the entropy (ΔS) of each adsorbed state is yielded from DFT calculation and ΔU is applied potential, whereas

the thermodynamic corrections for gas molecules are from standard tables. Norskov's computational hydrogen electrode is applied to calculate the reaction ΔG for OER[3]. In the method, with the standard conditions (pH = 0, P = 1 bar, T = 298 K), the ΔG of the reaction:

$$A^{*} + H^{+} + e^{-} \rightarrow AH^{*} \tag{2}$$

could be calculated from the reactions:

$$A^{*} + \frac{1}{2}H_2 \rightarrow AH^{*} \tag{3}$$

$$\Delta G = G(AH^{*}) - G(1/2H_2) - G(A^{*}) + e\Delta U \tag{4}$$

To determine the doping position, the segregation energy (E_{seg}) was calculated. E_{seg} is defined as the total energy difference of the alloy with dopant in the first layer and sub-layer[4]. The segregation energy is calculated by:

$$E_{seg} = E_{1st\text{-}layer} - E_{sub\text{-}layer} \tag{5}$$

where E_{seg} is the calculated segregation energy, $E_{1st\text{-}layer}$ represents the total energy of the alloy with the dopant in the first layer, and $E_{sub\text{-}layer}$ is the total energy of the alloy with the dopant in the sub-layer.

Finite element analysis

Finite element analysis numerical simulation was conducted using the same procedure according to the reference[5]. The positive charge density on the electrodes, and the OH^{-} ion density close to the electrode surface are simulated by using the COMSOL Multiphysics solver. The positive charge density on the electrode was solved by the "electric currents" module at 1.5 V. In this study, a CoO nanoneedle structure was selected as the model. The effects of different tip radii and surfaces on the electric field strength were investigated. Four models with different radii and disparate surfaces were constructed in which the surface structure and the radii of the tips were

constructed based on the SEM images. These models include a nanoneedle (top radius: 110 nm, bottom radius: 150 nm), a nanoneedle with a smooth surface (top radius: 3.2 nm, bottom radius: 60 nm), a nanoneedle (top radius: 3.2 nm, bottom radius: 60 nm) with 1.5 nm spheres on the surface. In addition, to explore the proximity effect, the 1.5 nm spheres with different distances (16 nm and 6 nm) were also constructed. The electrolyte conductivity was taken to be 10 S m^{-1}. The charge density ρ is calculated based on Gauss's law,

$$\rho = \varepsilon_r \varepsilon_0 \nabla \cdot E \tag{6}$$

where ε_0 represents the dielectric function for a vacuum, and ε_r represents the dielectric function of the materials, which are 78.5 for the electrolyte and 1 for the electrode.

The Gouy-Chapman-Stern model consisting of a Helmholtz layer and a diffusion layer was used to model the electrical double layer. The Helmholtz layer is composed of the surface-adsorbed OH^- ions on the electrode surface. The diffusion layer consists of both cations and anions. The Poisson-Nerst-Planck equations were solved in a steady state of dynamic equilibrium between electrostatic forces and diffusion[5]:

$$\nabla^2 V = \begin{cases} 0 & d < d_H \\ (c_k - c_{OH})F & d > d_H \end{cases} \tag{7}$$

$$V \cdot \left(D V_{ci} + \frac{D z_i e}{k_B T} C_i \nabla V \right) = 0 \tag{8}$$

where d is the distance from the electrode surface into the electrolyte, and d_H is the thickness of the Helmholtz layer, which is taken as the radius of an OH^- ion (0.137 nm)[6]. That is, $d < d_H$ within the Helmholtz layer, and $d > d_H$ in the diffusion layer. c_i with $i \in \{K^+, OH^-\}$ are the concentrations of K^+ or OH^- ion, z_i is the valences of ions, e is the elementary charge, k_B is Boltzmann constant, the absolute temperature T was

taken as 297.3 K. The diffusion coefficients D of K^+, OH^- and H^+ ions in water are 2.14×10^{-9} m^2 s^{-1}, 1.9×10^{-9} m^2 s^{-1} and 7.10×10^{-9} m^2 s^{-1}, respectively[7].

Electrochemical in situ Raman measurements

The surface-enhanced Raman spectroscopy was carried out on a confocal microscope Raman system (Renishaw InVia). A He-Ne laser with a 632.8 nm excitation wavelength and a 50× microscope objective with a numerical aperture of 0.55 was used in all Raman measurements. Electrochemical in situ Raman experiments were carried out in a homemade Raman cell with potential control on CHI660E. Before the test, the 55 nm Au@2 nm SiO_2 nanoparticles were prepared which were dropped on the catalysts as Raman-signal amplifiers. Specifically, the 55 nm Au nanospheres were first synthesized according to the following procedure in the previous report[8]. 1.4 mL of sodium citrate solution (1 wt.%) was quickly added into 200 mL of boiling $HAuCl_4$ solution (0.01 wt.%). Then the mixture was refluxed for 20 min to obtain the spherical nanoparticles with a diameter of 55 nm. Finally, the Au colloidal solution was cooled to room temperature in an ambient atmosphere for the preparation of Au @SiO_2 nanoparticles. For preparing the 55 nm Au@2 nm SiO_2 nanoparticles, 0.4 mL of (3-aminopropyl) trimethoxysilane solution (1 mM) was dropwise added into 30 mL of the as-prepared Au solution under stirring at room temperature. Next, 3.2 mL of sodium silicate solution (0.54 wt.%) was quickly added to the solution, then transferred to a water bath and stirred for 30 min at 99 °C. Finally, 1.5 mL of the hot solution was added into a centrifuge tube and immediately immersed it in an ice bath to stop the reaction. The solution was centrifuged at 4500 rpm and washed twice with Milli-Q water for the Raman measurements.

Oxygen isotope experiment

The oxygen isotope labelling experiments were conducted in a three-step approach

(Fig. 3f)[9,10]. First, the as-prepared Fe, F-CoO NNAs catalyst was labelled in 0.1 M KOH $H_2^{18}O$ aqueous solution (denoted as ^{18}O-KOH) using a chronoamperometry method for 20 min at 1.664 V vs. RHE and then rinsed by water. Subsequently, the ^{18}O-KOH electrolyte was replaced with 0.1 M KOH $H_2^{16}O$ (^{16}O-KOH) aqueous solution. At last, in situ Raman spectroscopy and on-line differential electrochemical mass spectrometry (DEMS) measurements were conducted to directly identify the participation of lattice oxygen during the OER. Specifically, in situ Raman spectroscopy was conducted on the Fe, F-$Co^{18}O$ NNAs sample from 1.23 to 1.43 V vs. RHE in ^{16}O-KOH electrolytes. While DEMS was conducted to detect the O_2 gas generated during the OER of Fe, F-$Co^{18}O$ NNAs sample from 1.23 to 2.23 V vs. RHE.

2. Supplementary Figures

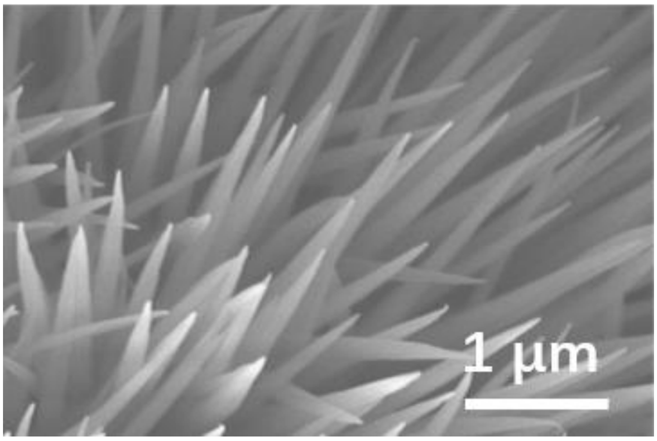

Supplementary Fig. 1. FESEM image of the Co(OH)F NNAs.

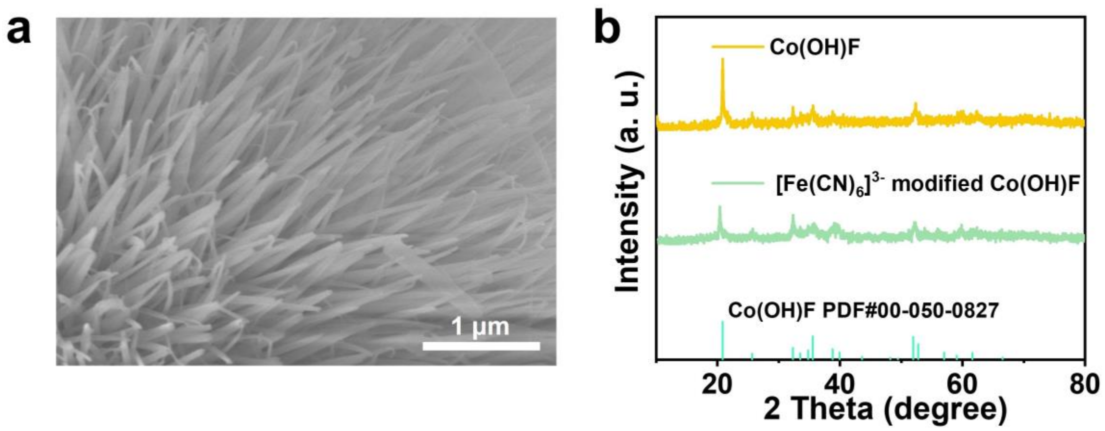

Supplementary Fig. 2. a FESEM image of the $Fe[(CN)_6]^{3-}$ modified Co(OH)F NNAs. **b** XRD patterns of the Co(OH)F NNAs and the $Fe[(CN)_6]^{3-}$ modified Co(OH)F NNAs.

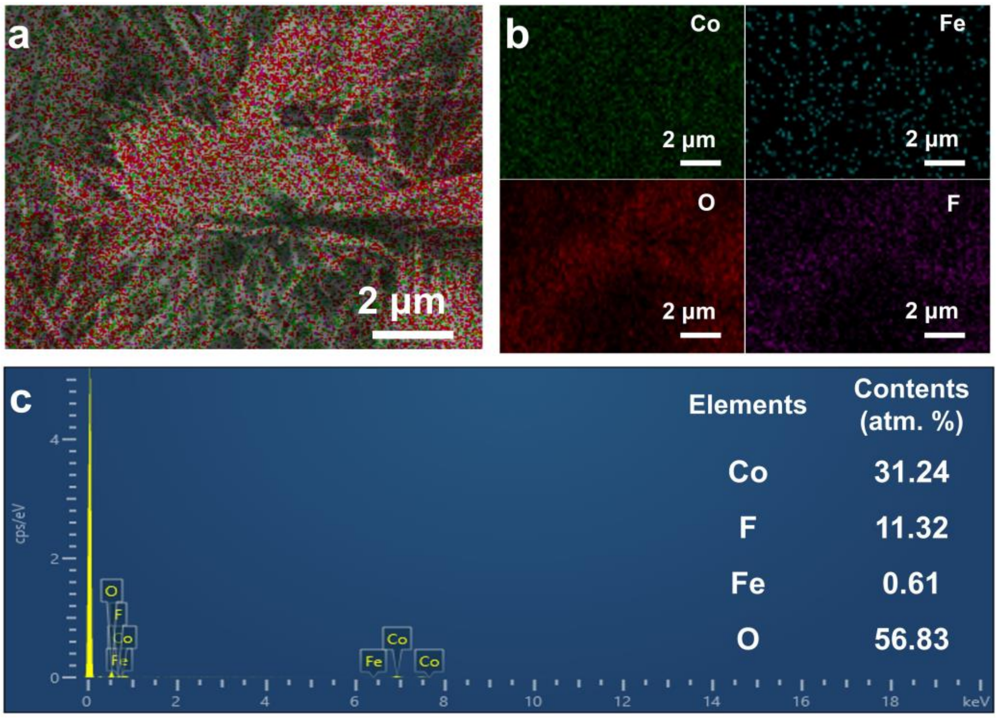

Supplementary Fig. 3. (**a**) SEM image and (**b**) corresponding EDS mapping images, and (**c**) element contents in Fe, F-CoO NNAs.

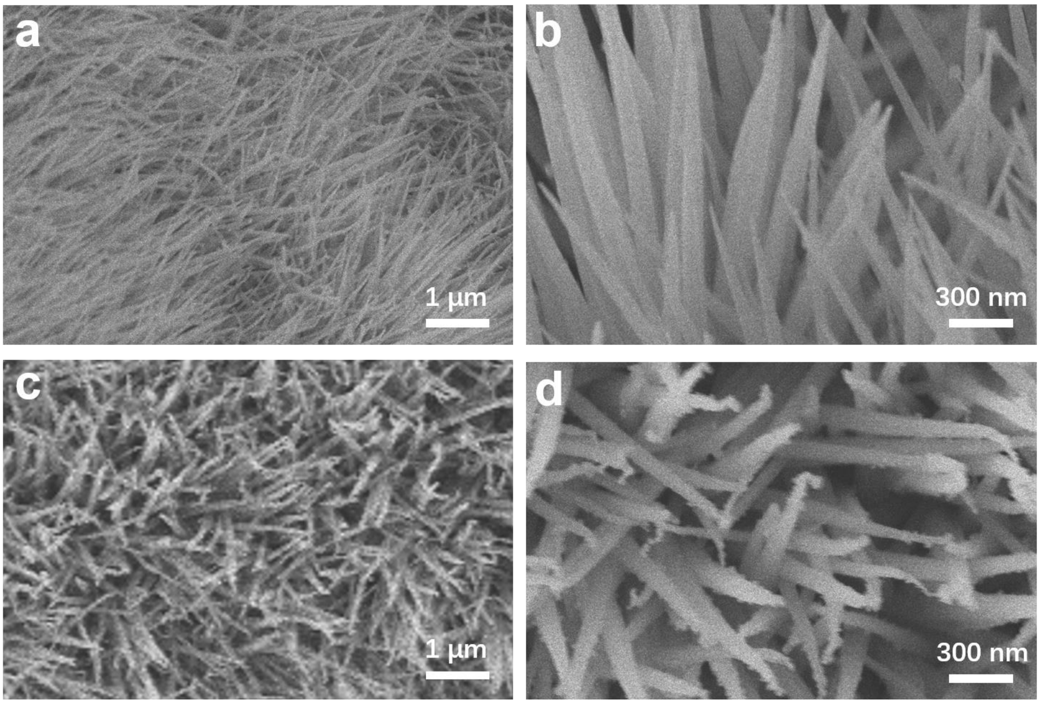

Supplementary Fig. 4. FESEM images of the Fe, F-CoO NNAs prepared with different concentrations of $K_3[Fe(CN)_6]$: (**a, b**) 0.005 M and (**c, d**) 0.02 M.

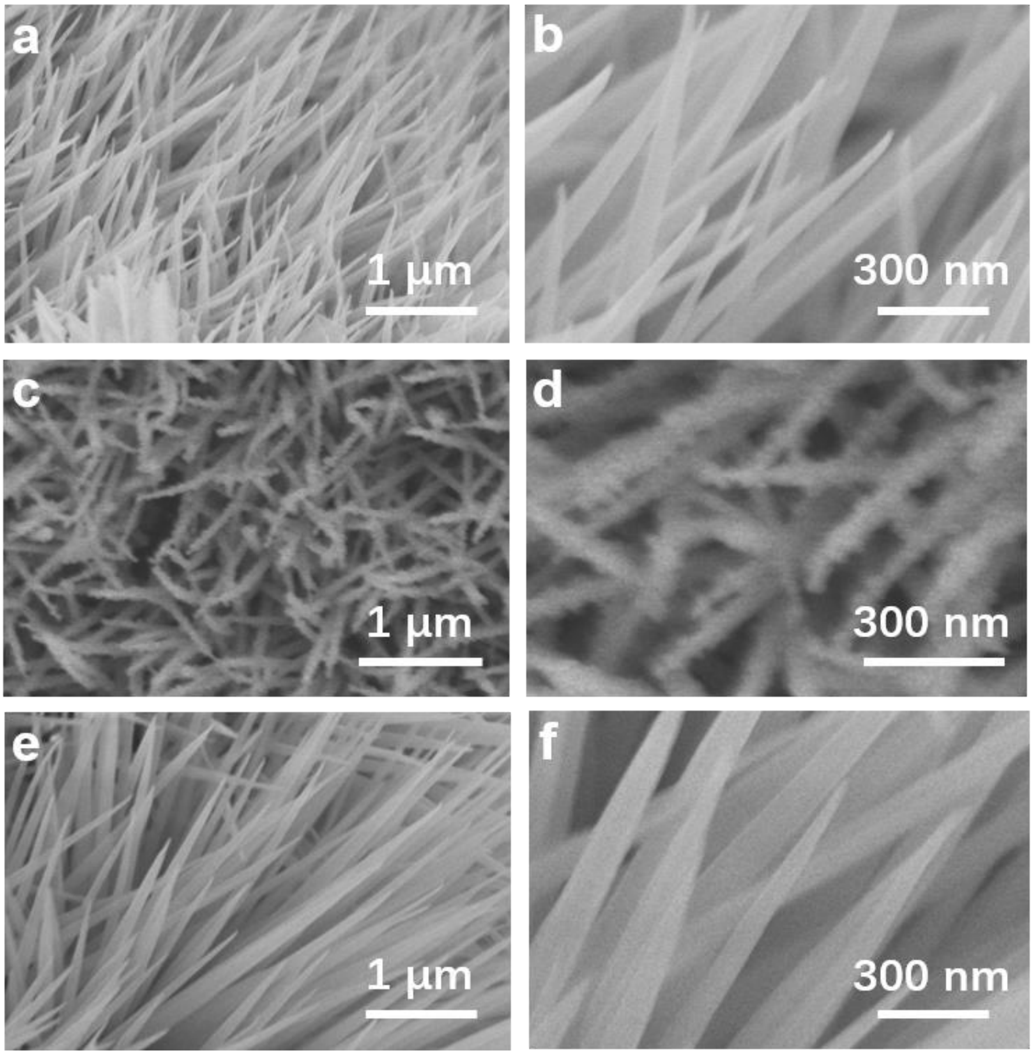

Supplementary Fig. 5. FESEM images of the (**a, b**) CoO NNAs, (**c, d**) Fe-CoO NNAs, (**e, f**) F-CoO NNAs.

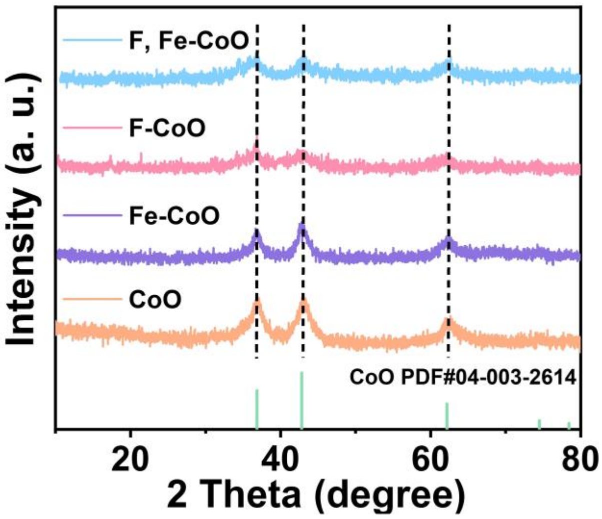

Supplementary Fig. 6. XRD patterns of the Fe, F-CoO NNAs, Fe-CoO NNAs, F-CoO NNAs, and CoO NNAs.

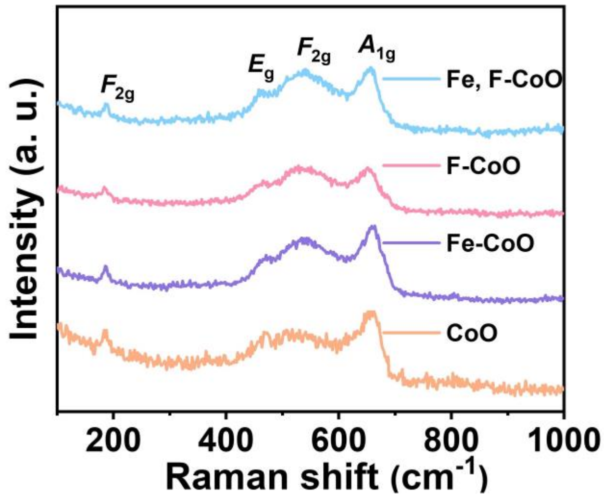

Supplementary Fig. 7. Raman spectra of the Fe, F-CoO NNAs, Fe-CoO NNAs, F-CoO NNAs, and CoO NNAs.

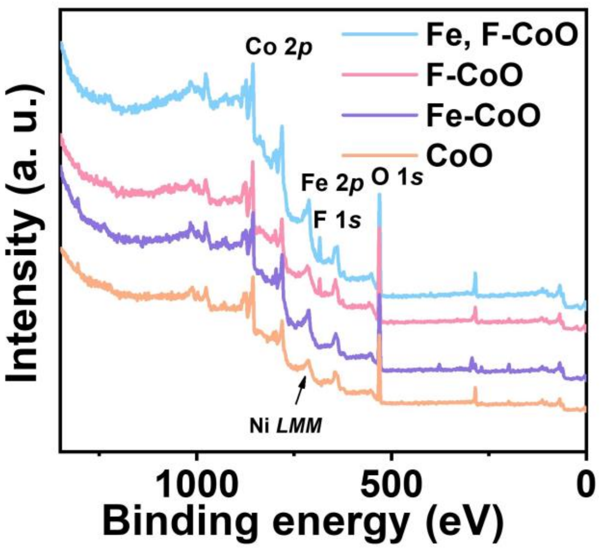

Supplementary Fig. 8. XPS survey spectra of the Fe, F-CoO NNAs, F-CoO NNAs, Fe-CoO NNAs, and CoO NNAs.

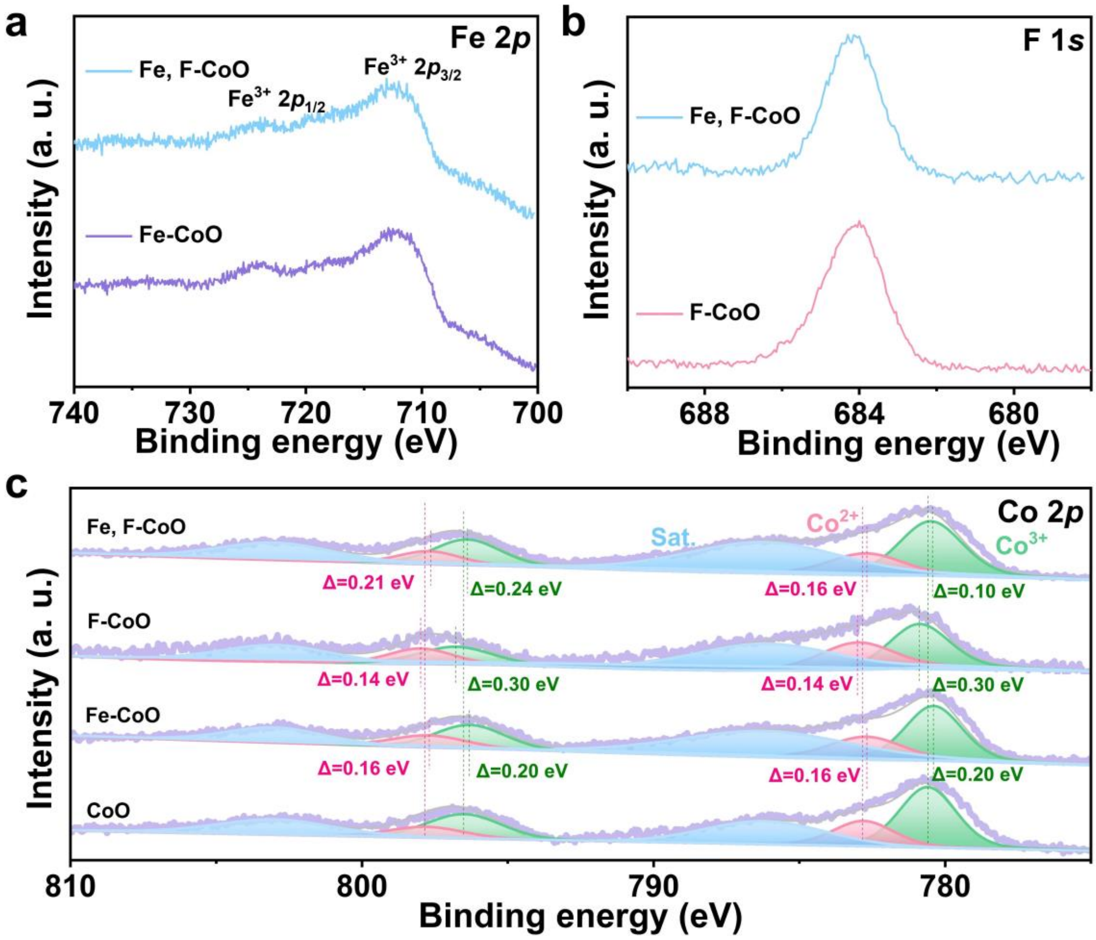

Supplementary Fig. 9. **a** High-resolution Fe 2p XPS spectra of the Fe, F-CoO NNAs, and Fe- CoO NNAs. **b** High-resolution F 1s XPS spectra of the Fe, F-CoO NNAs, and F-CoO NNAs. **c** High-resolution Co 2p XPS spectra of the Fe, F-CoO NNAs, F-CoO NNAs, Fe-CoO NNAs, and CoO NNAs.

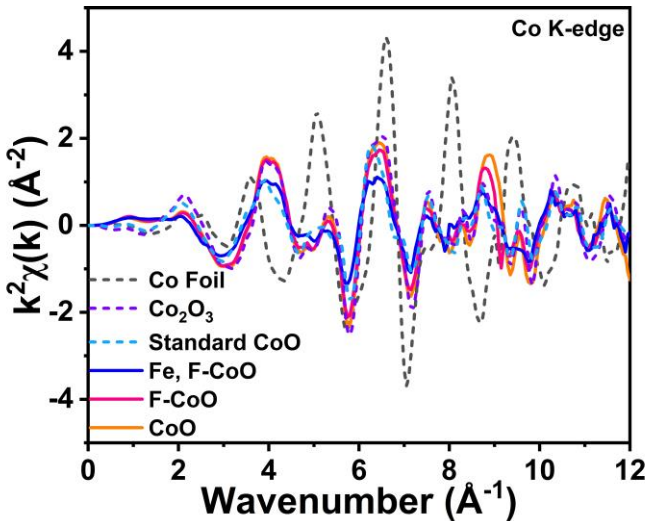

Supplementary Fig. 10. Co K-edge EXAFS oscillation functions of CoO NNAs, F-CoO NNAs, Fe, F-CoO NNAs, and the standard references including Co foil, standard CoO and Co_2O_3.

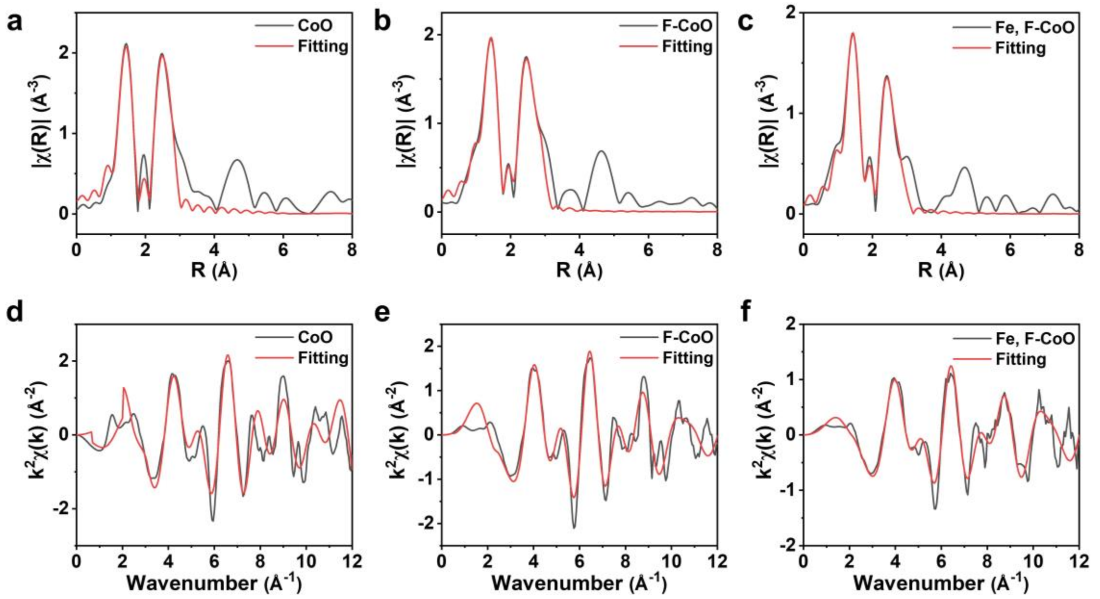

Supplementary Fig. 11. Co K-edge EXAFS fitting results for the (**a, d**) CoO NNAs, (**b, e**)F-CoO NNAs, and (**c, f**) Fe, F-CoO NNAs.

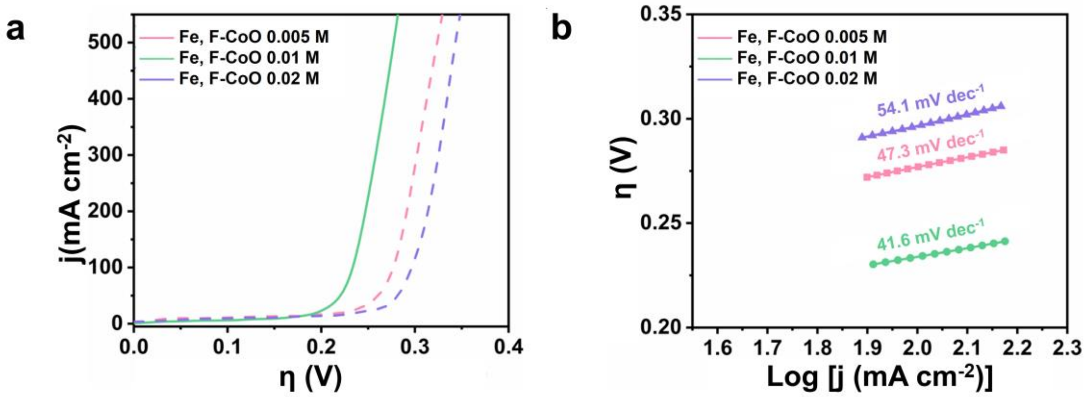

Supplementary Fig. 12. (**a**) Polarization curves and (**b**) Tafel plots of the Fe, F-CoO NNAs prepared with 0.005 M, 0.01 M, and 0.02 M $K_3[Fe(CN)_6]$ solution.

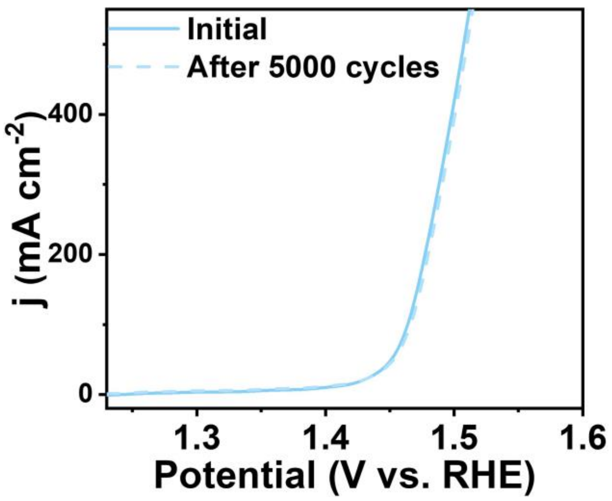

Supplementary Fig. 13. LSV curves of the Fe, F-CoO NNAs before and after 5000 CV cycles with the potential window of 1−2.05 V.

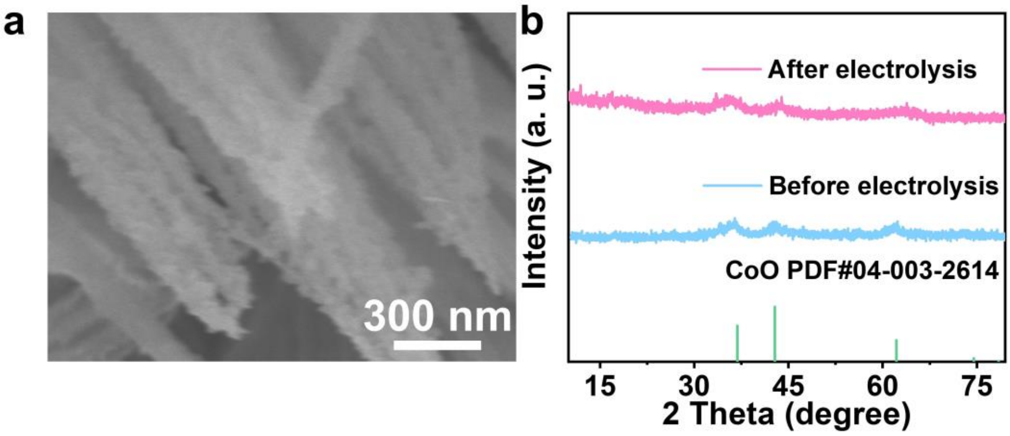

Supplementary Fig. 14. a FESEM image of the Fe, F-CoO NNAs after cycling test. **b** XRD patterns of the Fe, F-CoO NNAs before and after cycling test.

.

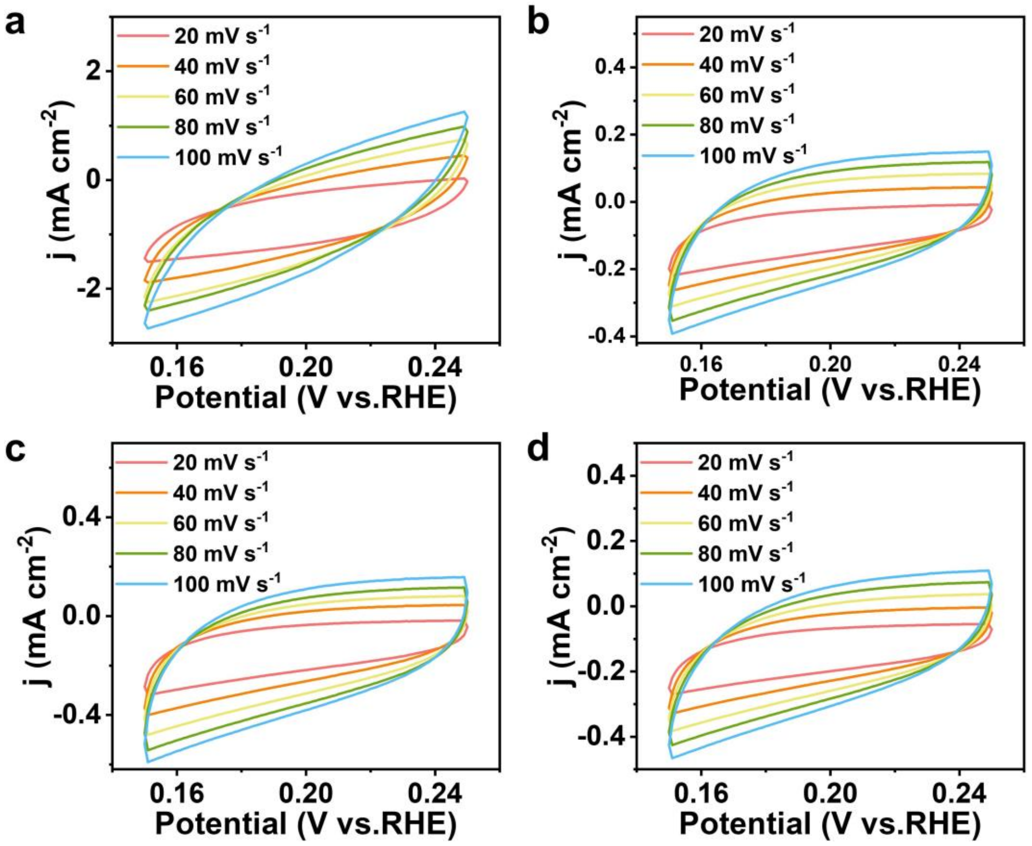

Supplementary Fig. 15. CV profiles of (**a**) Fe, F-CoO NNAs, (**b**) F-CoO NNAs, (**c**) Fe-CoO NNAs, and (**d**) CoO NNAs at 20−100 mV s^{-1} in the potential range of 0.15−0.25 V.

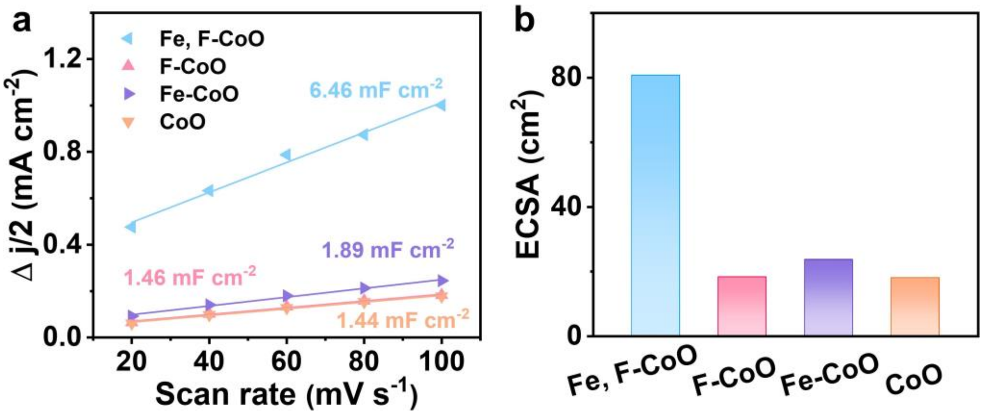

Supplementary Fig. 16. a The corresponding current densities (Δj/2) as a function of scan rates (*v*) of the Fe, F-CoO NNAs, F-CoO NNAs, Fe-CoO NNAs, and CoO NNAs. **b** ECSAs of the Fe, F-CoO NNAs, F-CoO NNAs, Fe-CoO NNAs, and CoO NNAs.

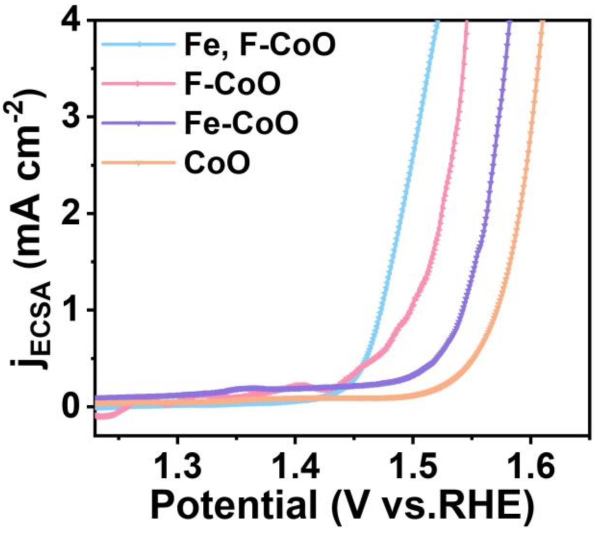

Supplementary Fig. 17. The ESCA-normalized polarization curves of the Fe, F-CoO NNAs s, F-CoO NNAs, Fe-CoO NNAs, and CoO NNAs.

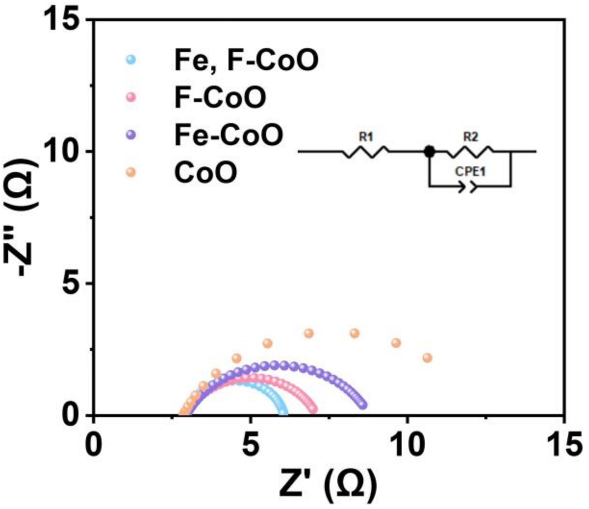

Supplementary Fig. 18. Nyquist plots of the Fe, F-CoO NNAs, F-CoO NNAs, Fe-CoO NNAs, and CoO NNAs with an inset of the equivalent circuit diagram.

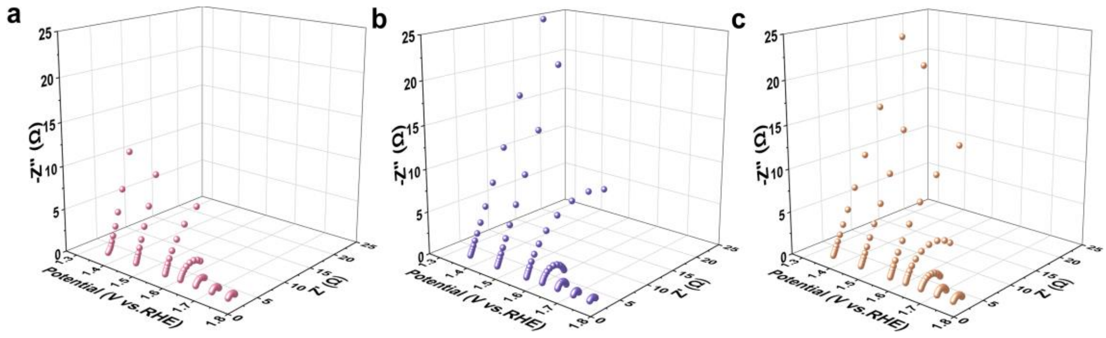

Supplementary Fig. 19. In situ EIS spectra of (**a**) F-CoO NNAs, (**b**) Fe-CoO NNAs, and (**c**) CoO NNAs at various potentials.

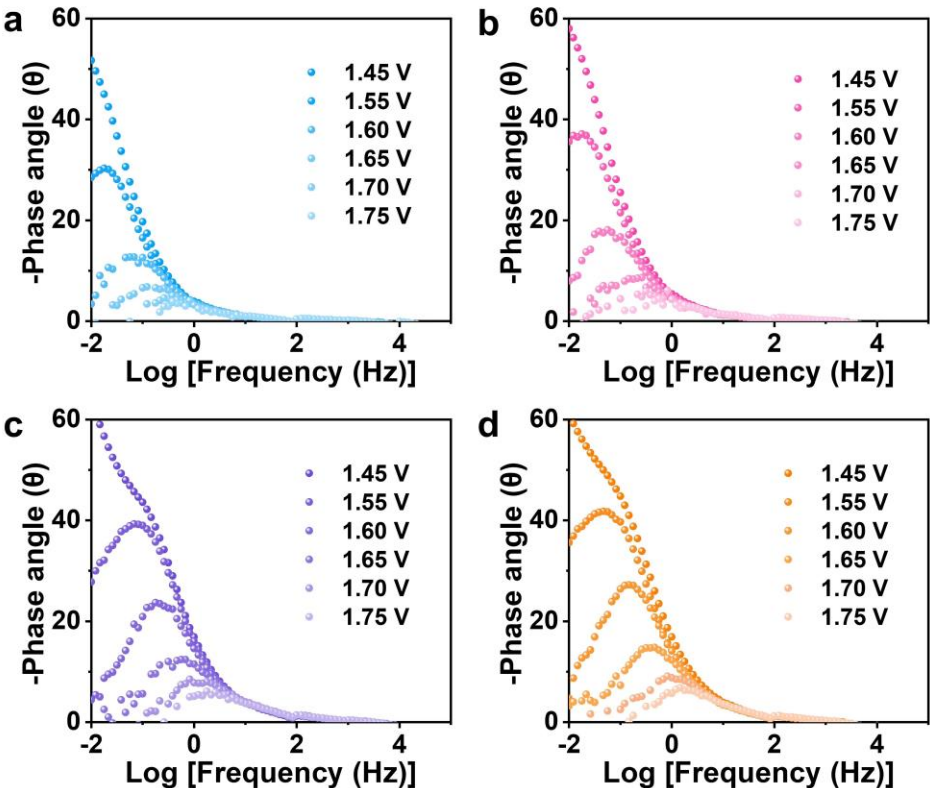

Supplementary Fig. 20. Bode phase plots of (**a**) Fe, F-CoO NNAs, (**b**) F-CoO NNAs, (**c**) Fe-CoO NNAs, and (**d**) CoO NNAs at various potentials.

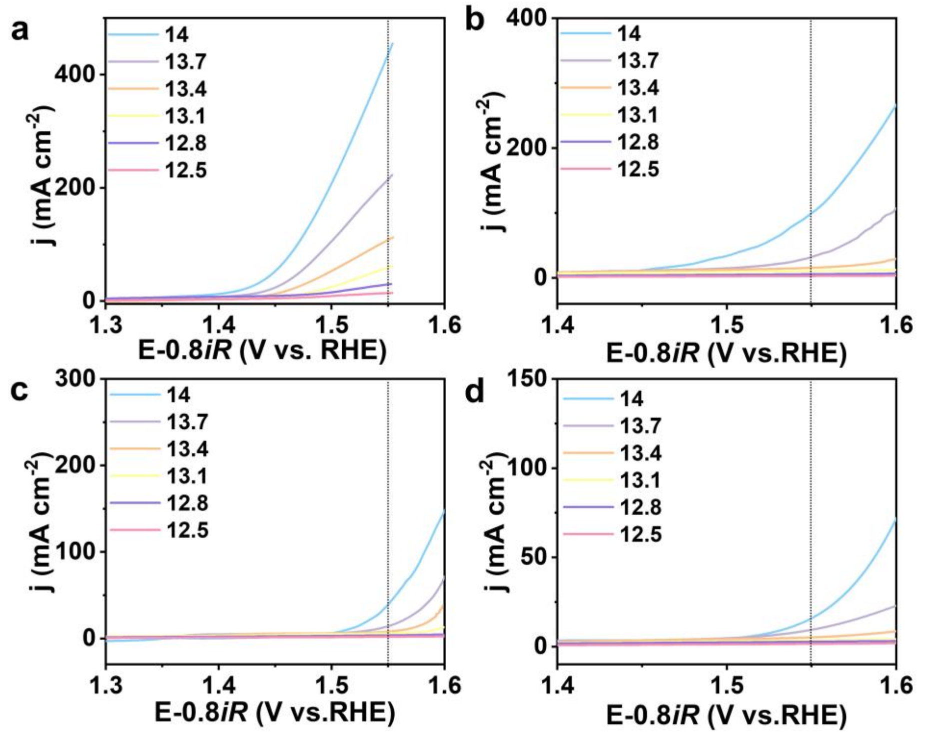

Supplementary Fig. 21. pH dependence on OER activity. The polarization curves of (**a**) Fe, F- CoO NNAs, (**b**) F-CoO NNAs, (**c**) Fe-CoO NNAs, and (**d**) CoO NNAs in various pH conditions.

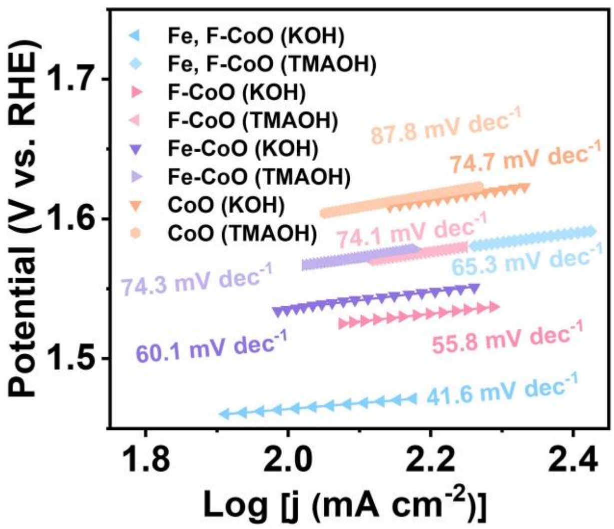

Supplementary Fig. 22. Corresponding Tafel slopes of the Fe, F-CoO NNAs, F-CoO NNAs, Fe-CoO NNAs, and CoO NNAs in 1 M KOH and 1 M TMAOH electrolytes.

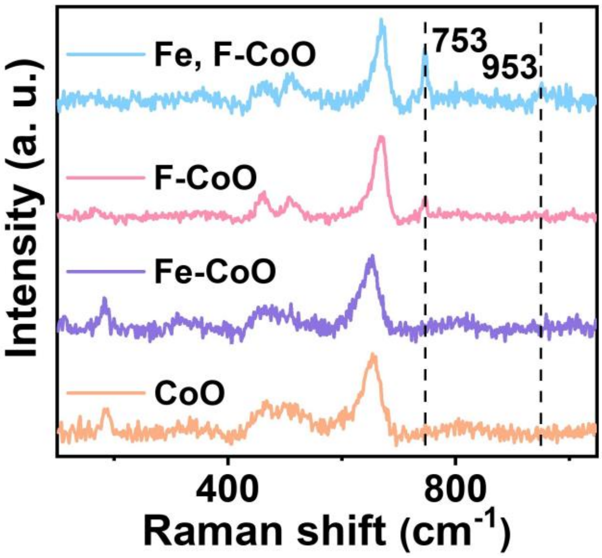

Supplementary Fig. 23. Raman spectra of the Fe, F-CoO NNAs, F-CoO NNAs, Fe-CoO NNAs, and CoO NNAs in 1 M TMAOH solution after chronoamperometry under 1.55 V vs RHE for 30 min.

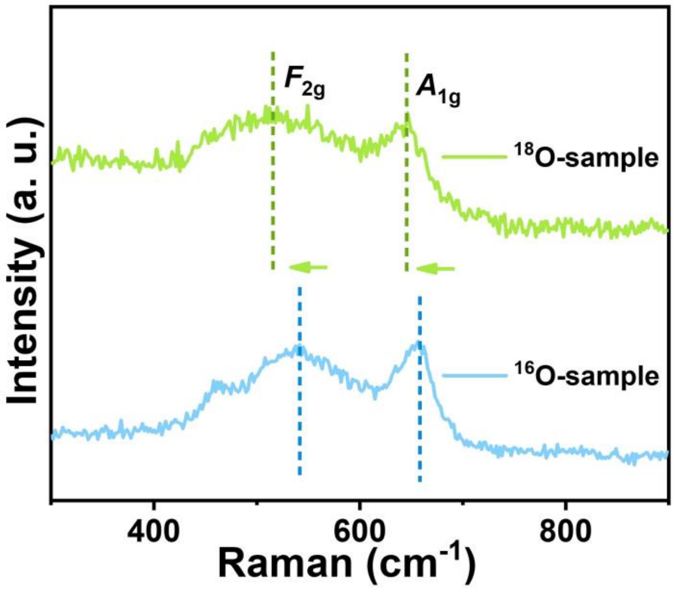

Supplementary Fig. 24. Raman spectra of the pristine Fe, F-CoO NNAs and Fe, F-$Co^{18}O$ NNAs samples.

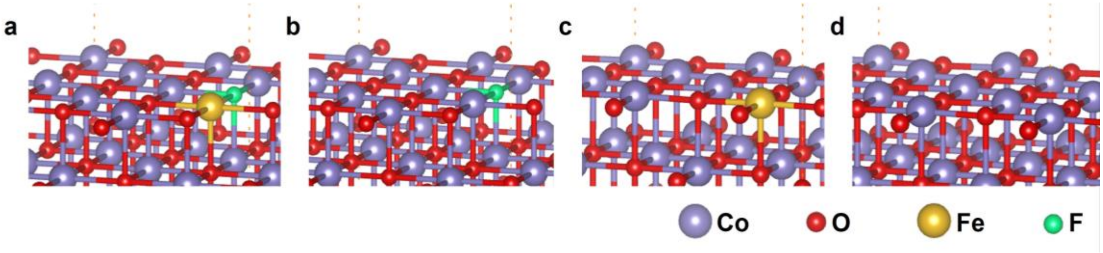

Supplementary Fig. 25. Theoretical calculation models of (**a**) Fe, F-CoO, (**b**) F-CoO, (**c**) Fe-CoO, and (**d**) CoO slabs.

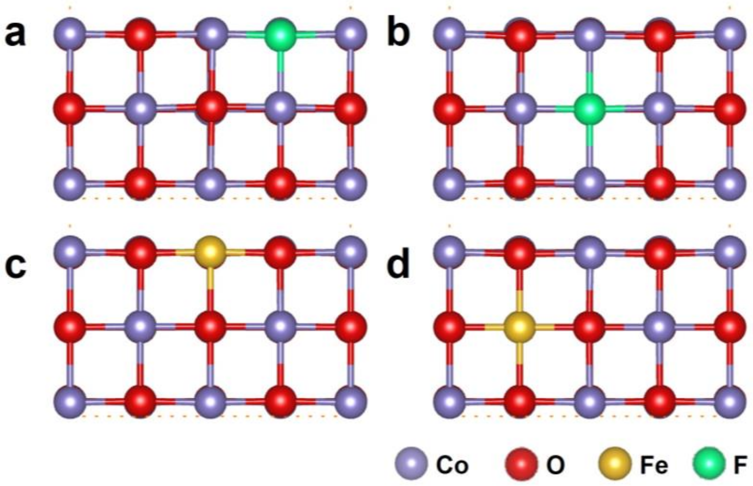

Supplementary Fig. 26. Slab models for (**a**) F dopant within the first layer of CoO, (**b**) F dopant within the sub-layer of CoO, (**c**) Fe dopant within the first layer of CoO, and (**d**) Fe dopant within the sub-layer of CoO.

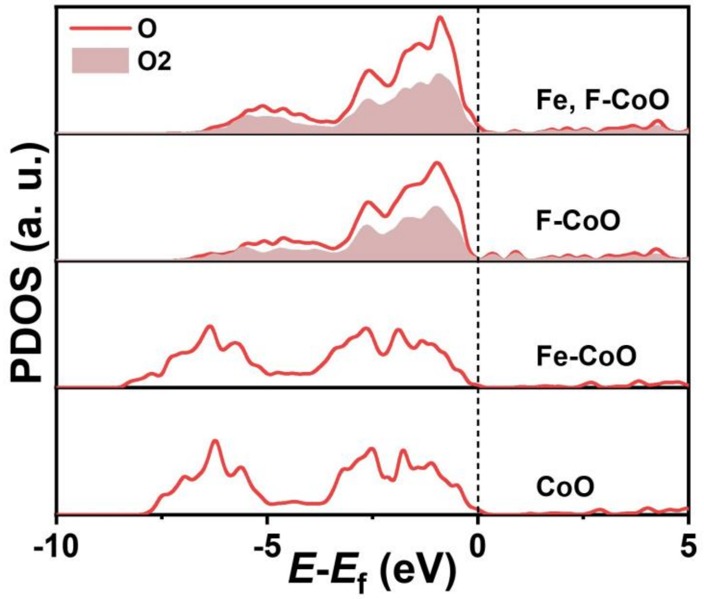

Supplementary Fig. 27. PDOS of O 2*p* orbits and non-bonding oxygen states (O2) in CoO, Fe-CoO, F-CoO, and Fe, F-CoO slabs.

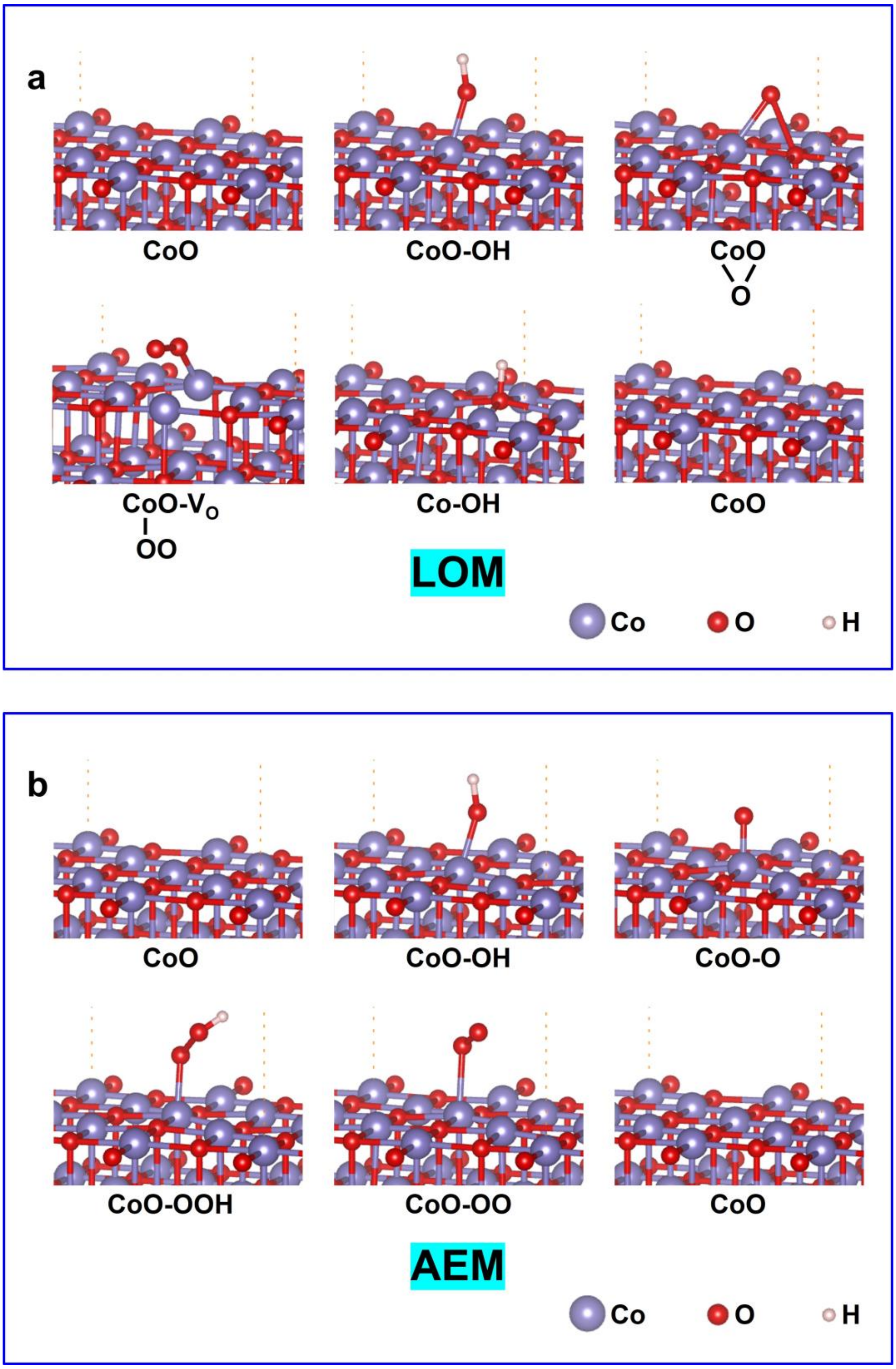

Supplementary Fig. 28. Models of the different intermediates in (**a**) LOM and (**b**) AEM pathways on the CoO slab.

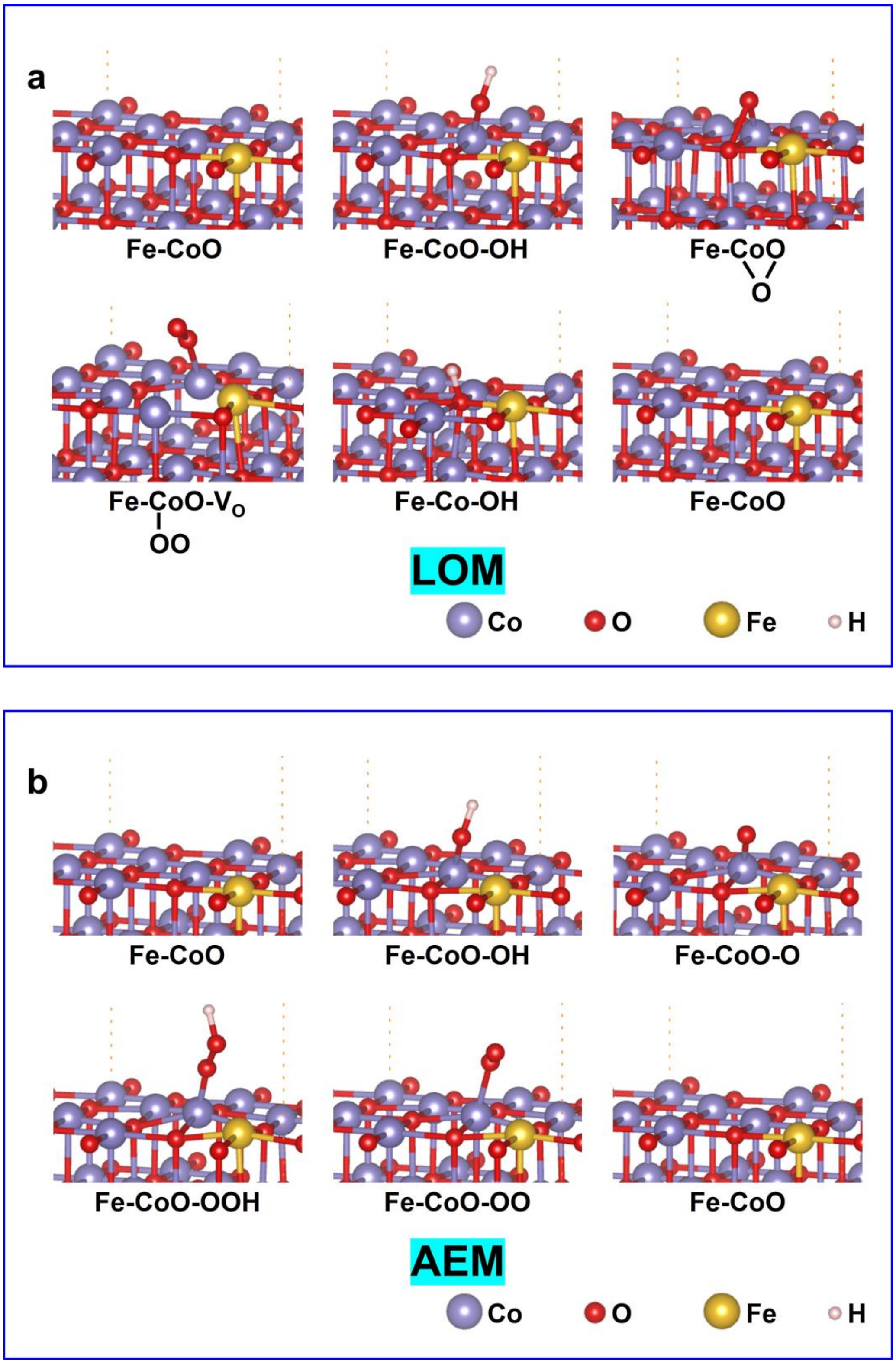

Supplementary Fig. 29. Models of the different intermediates in (**a**) LOM and (**b**) AEM pathways on the Fe-CoO slab.

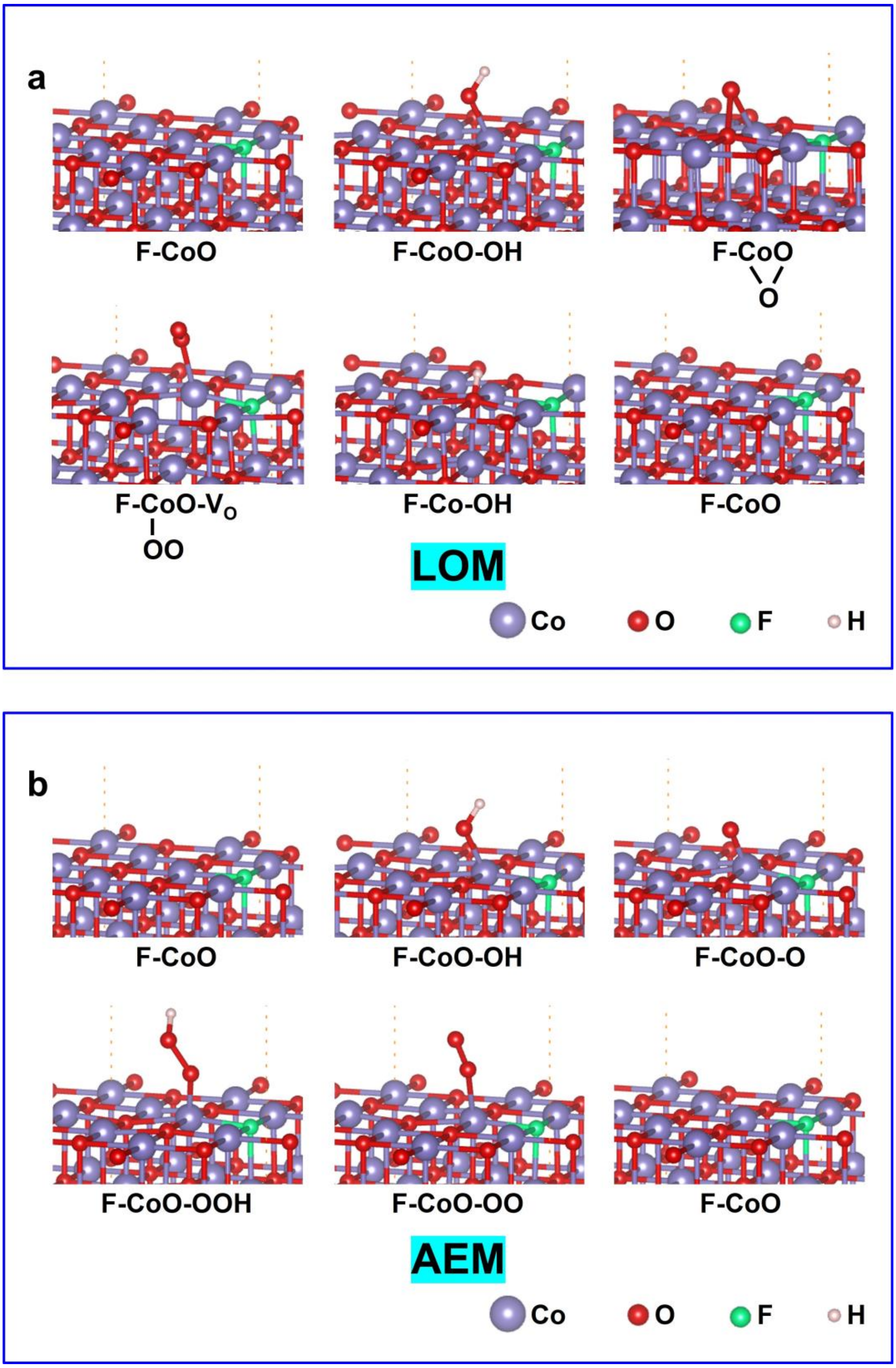

Supplementary Fig. 30. Models of the different intermediates in (**a**) LOM and (**b**) AEM pathways on the F-CoO slab.

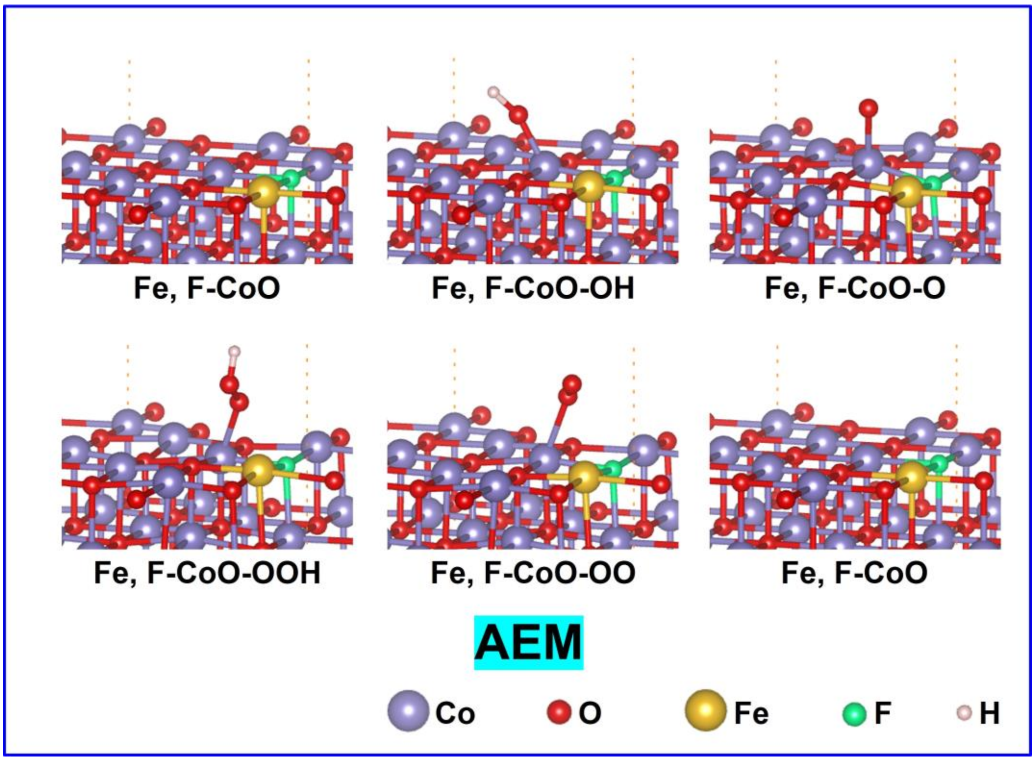

Supplementary Fig. 31. Models of the different intermediates in AEM pathways on the Fe, F-CoO slab.

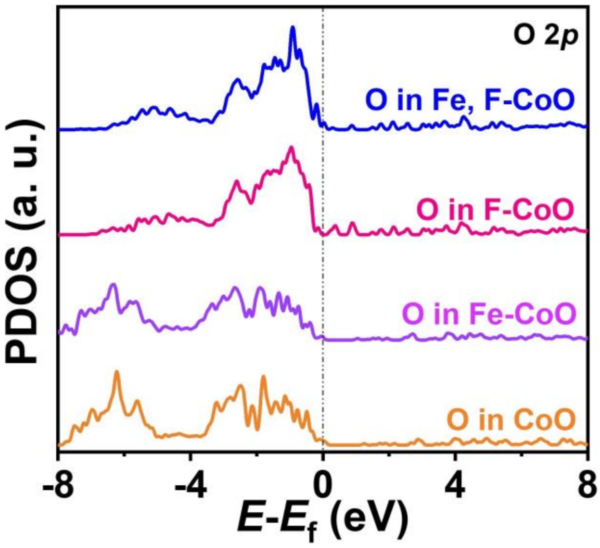

Supplementary Fig. 32. Calculated PDOSs of O 2*p* orbits in the Fe, F-CoO, F-CoO, Fe-CoO, and CoO slabs.

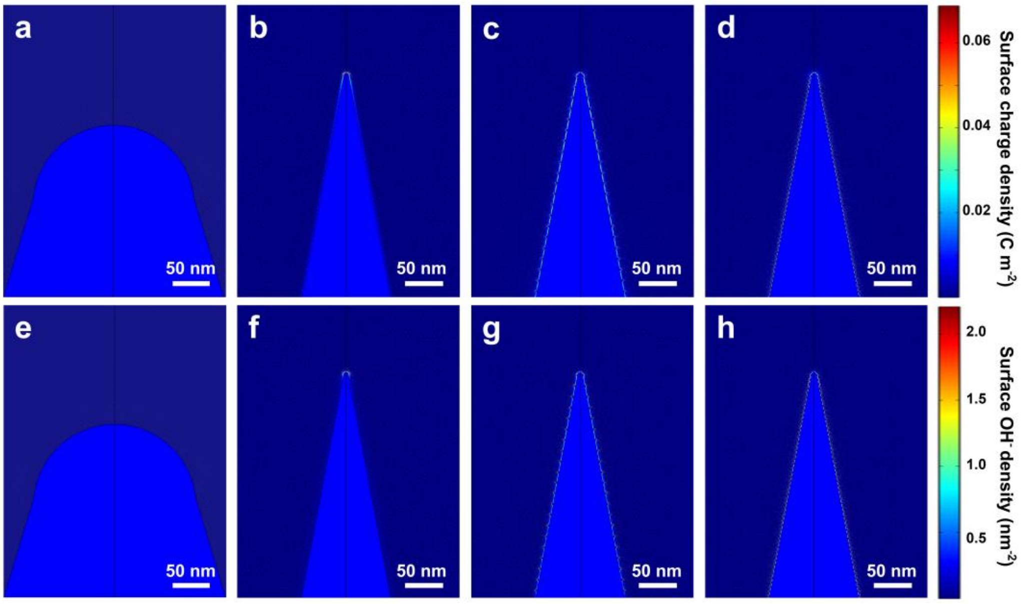

Supplementary Fig. 33. Finite element simulations. (**a-d**) Surface positive charge density distribution and (**e-h**) surface OH^- density distribution on different electrode models. (**a, e**) The nanoneedles with a top radius of 110 nm and a bottom radius of 150 nm. (**b, f**) The nanoneedles with a top radius of 3.2 nm and a bottom radius of 60 nm. The nanoneedles (top radius: 3.2 nm and bottom radius: 60 nm) with 1.5 nm spherules on the surface, and the gaps between the spherules are (**c, g**) 16 nm and (**d, h**) 6 nm.

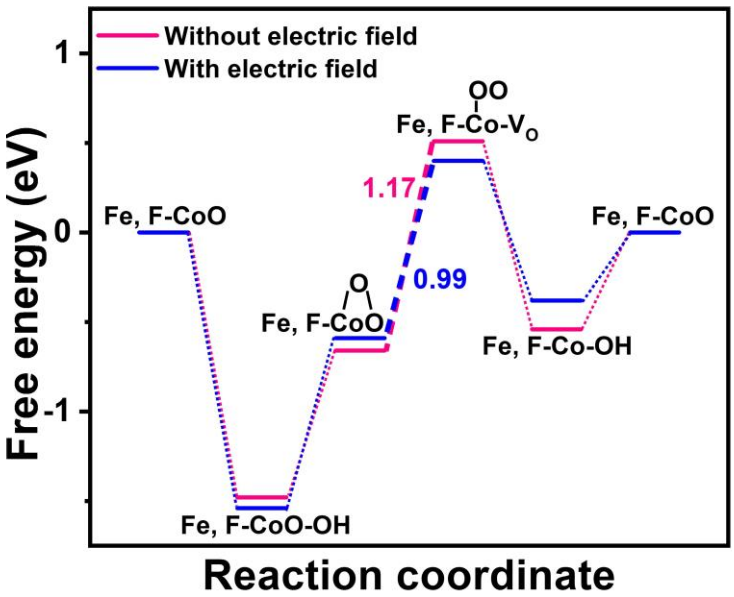

Supplementary Fig. 34. The Gibbs free energy diagrams of OER steps via LOM pathway on Fe, F-CoO slab without and with an electric field (1.58×10^{-2} V/Å).

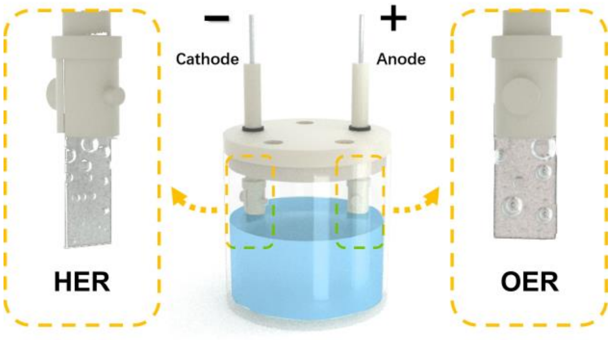

Supplementary Fig. 35. Schematic illustration of the alkaline electrolyzer in which HER occurs on the cathode and OER occurs on the anode.

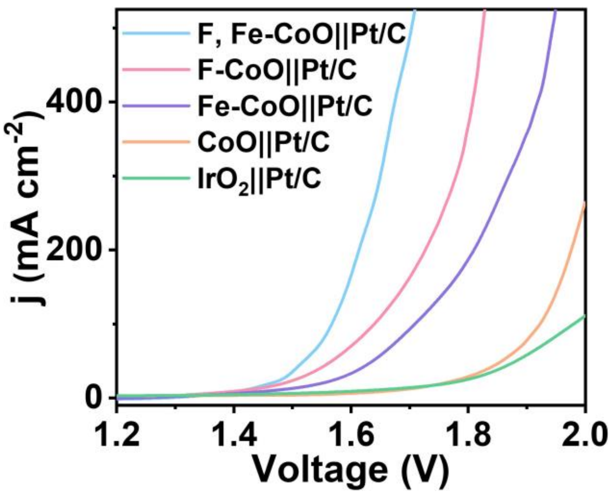

Supplementary Fig. 36. Polarization curves of the alkaline electrolyzers based on the Fe, F-CoO NNAs, F-CoO NNAs, Fe-CoO NNAs, CoO NNAs and IrO_2.

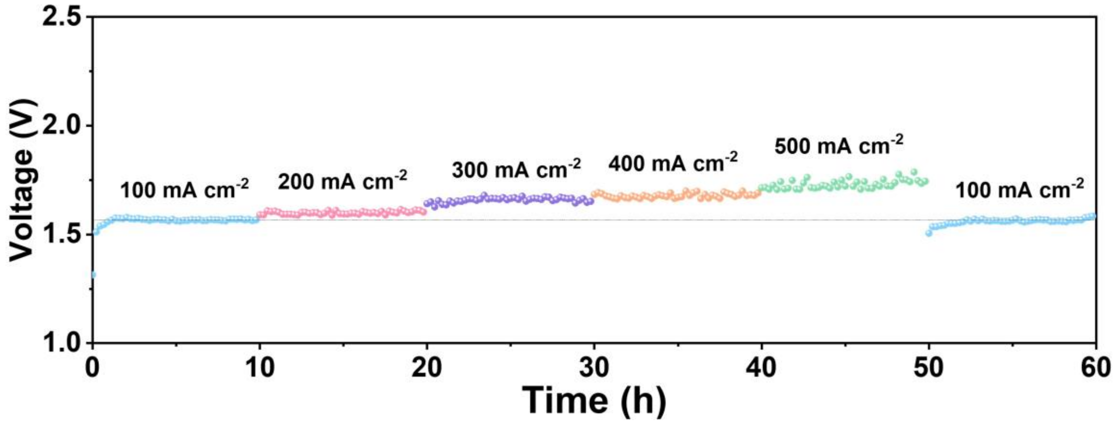

Supplementary Fig. 37. Long-term stability of the Fe, F-CoO NNAs||Pt/C electrolyzer at multistep current densities from 100 to 500 mA cm^{-2}.

3. Supplementary Tables

Supplementary Table 1. The ratios of the various types of oxygen species in the Fe, F-CoO NNAs, F-CoO NNAs, Fe-CoO NNAs, and CoO NNAs.

Catalysts	Lattice-O (%)	Hydroxyl (%)	Adsorbed H_2O (%)
Fe, F-CoO	49.9	44.4	5.7
F-CoO	10.1	82.8	7.1
Fe-CoO	35.1	57.5	7.4
CoO	27.3%	65.4%	7.3%

Supplementary Table 2. Structural parameters of CoO NNAs, F-CoO NNAs and Fe, F-CoO NNAs extracted from the EXAFS fitting. (S_0^2=0.80).

Scattering pair		CN	R (Å)	σ^2 (10^{-3}Å^2)	ΔE_0 (eV)	R factor
CoO	Co-O	4.0±0.1	2.13±0.02	5.3±0.7	4.5±0.3	0.02
	Co-Co	5.1±0.9	3.01±0.02	5.6±0.9	-4.2±0.5	0.02
F-CoO	Co-O/F	3.9±0.5	2.13±0.02	6.4±0.7	4.5±0.5	0.02
	Co-Co	5.2±0.7	3.01±0.02	6.8±0.9	-4.2±0.5	0.02
Fe, F-CoO	Co-O/F	4.1±0.2	2.13±0.02	6.9±0.7	4.5±0.5	0.02
	Co-Co/Fe	5.1±0.5	3.01±0.02	7.7±0.3	-4.2±0.5	0.02

S_0^2 is the amplitude reduction factor (S_0^2=0.8); CN is the coordination number; R is the interatomic distance (the bond length between central atoms and surrounding coordination atoms); σ^2 is Debye-Waller factor (a measure of thermal and static disorder in absorber-scatterer distances); ΔE_0 is edge-energy shift (the difference between the zero kinetic energy value of the sample and that of the theoretical model). R factor is used to value the goodness of the fitting.

Supplementary Table 3. Comparison of the overpotentials of Fe, F-CoO NNAs with the state-of-the-art transition metal-based OER electrocatalysts at various current densities (10 mA cm^{-2}, 100 mA cm^{-2}, and 500 mA cm^{-2}).

Catalysts	η_{10} (mV)	η_{100} (mV)	η_{500} (mV)	References
Fe, F-CoO NNAs	169	234	277	This work
LSC/K-$MoSe_2$	230	/	/	Nat. Commun. 2021, 12, 4606.
S-FeOOH/IF	244	308	/	Adv. Funct. Mater. 2022, 2112674
Fe-doped-(Ni-MOFs)/FeOOH	/	278	/	Angew. Chem. Int. Ed. 2022, 61, e202116934.
CF-FeSO	192	230	/	Nat. Commun. 2022, 13, 605.
$CoSe_2$–D_{Fe}–V_{Co}	/	520	/	Nat. Commun. 2020, 11, 1664.
W-$NiS_{0.5}Se_{0.5}$	171	239	/	Adv. Mater. 2022, 34, 2107053.
CoFe-LDH/GF	252	285	/	Chin. Chem. Lett. 2022, 33, 890-892.
V−CoP_2/CC	91	498	/	Angew. Chem. Int. Ed. 2022, 61, e202116233.
Fe0.4Ni0.6-alloy fiber paper	/	287	/	Appl. Catal. B: Environ. 2021, 286, 119902.
CF/VMFO	/	/	225	Nat. Commun. 2021, 12, 1380.
FeNi(VO_4)x@NF	/	274	/	Small 2020, 16, 2002412
NiMoFeO@NC	/	290	/	Matter 2020, 3, 2124-2137.
$Fe_{0.052}$Ni-POMo	255.3	295	/	Adv. Funct. Mater. 2021, 31, 2101792
NiFe-Boride	167	/	/	Nat. Commun. 2021, 12, 6089.
$Ni_xFe_yCo_{6-x-y}Mo_6C$/NF	212	275	336	Appl. Catal. B: Environ. 2021, 290, 120049.
NiFeB	/	252	/	Nat. Commun. 2022, 13, 6094.
NiMo-Fe	217	264	/	Appl. Catal. B: Environ. 2022, 307, 121150.
NFO-S5	/	232	/	Appl. Catal. B: Environ. 2022, 305, 121030
(Ni,Co)Se_2-GA	250	340	/	ACS Catal. 2017, 7, 6394.
Fe-CoP/NF	190	/	295	Adv. Sci. 2018, 5, 1800949
EBP/CoFeB	227	313	/	ACS Nano 2021, 15, 12418−12428

Supplementary Table 4. Comparison of the Tafel slope values of Fe, F-CoO NNAs with the state-of-the-art transition metal-based OER electrocatalysts.

Catalysts	Tafel slopes (mV dec^{-1})	References
Fe, F-CoO NNAs	41.6	This work
$Fe_{0.4}Ni_{0.6}$-alloy fiber paper	67	Appl. Catal. B: Environ. 2021, 286, 119902.
S-FeOOH/IF	59	Adv. Funct. Mater. 2022, 2112674
Fe-doped-(Ni-MOFs)/FeOOH	50	Angew. Chem. Int. Ed. 2022, 61, e202116934
W-$NiS_{0.5}Se_{0.5}$	41	Adv. Mater. 2022, 34, 2107053.
CoFe-LDH/GF	61	Chin. Chem. Lett. 2022, 33, 890-892.
V-CoP_2/CC	40	Angew. Chem. Int. Ed. 2022, 61, e202116233
$FeNi(VO_4)_x$@NF	56.6	Small 2020, 16, 2002412
NiMoFeO@NC	66.6	Matter 2020, 3, 2124-2137.
$Fe_{0.052}$Ni-POMo	43.8	Adv. Funct. Mater. 2021, 31, 2101792
$Ni_xFe_yCo_{6-x-y}Mo_6C$/NF	55.1	Appl. Catal. B: Environ. 2021, 290, 120049.
$CoSe_2$-D_{Fe}-V_{Co}	53.5	Nat. Commun. 2020, 11, 1664.

Supplementary Table 5. Comparison of the electrocatalytic stability of Fe, F-CoO NNAs with the reported transition metal-based OER electrocatalysts at large current densities.

Catalysts	Stability	Current density (mA cm^{-2})	References
Fe, F-CoO	300 h	100	This work
	300 h	500	
NiFeB	130 h	500	Nat. Commun. 2022, 13, 6094.
CF/VMFO	30 h	250	Nat. Commun. 2021, 12, 1380.
NiMoN/NiFe LDH	250 h	1000	Nat. Commun. 2023, 14, 1873.
MIL-53(Fe)-2OH	100 h	100	Adv. Mater. 2023, 35, 2208904.
CoFePS	42 h	500	Adv. Funct. Mater. 2023, 2308422.
$FeCoSn(OH)_6$-300	200 h	100	Nat. Commun. 2022, 133, 1187.
SO-NFO NS_{D21}	24 h	100	ACS Energy Lett. 2023, 8, 3504.
$Co(OH)(CO_3)_{0.5}$	30 h	100	ACS Catal. 2023, 13, 8821.
Zn, S-Fe_3O_4-FeOOH/IF	100 h	500	Adv. Funct. Mater. 2023, 2303776.
Ni-BDC-1R	100 h	100	Angew. Chem. Int. Ed. 2022, 134, e202214794.
(Act)-(Ni,Mn)-$(Co)_{tet}(Co2)_{oct}O_4$ NSs	100 h	100	Angew. Chem. Int. Ed. 2023, 62, e202214600.

Supplementary Table 6. Corresponding fitted parameters of R_s and R_{ct} based on the Nyquist plots of Fe, F-CoO NNAs, F-CoO NNAs, Fe-CoO NNAs, and CoO NNAs.

Catalysts	R_s (Ω)	R_{ct} (Ω)
Fe, F-CoO	3.0	3.1
F-CoO	2.9	4.2
Fe-CoO	2.9	5.8
CoO	2.9	9.5

Supplementary Table 7. Energy barriers of the PDS in LOM and AEM pathways over Fe, F-CoO NNAs, F-CoO NNAs, Fe-CoO NNAs, and CoO NNAs.

Catalysts	Energy barrier of the PDS in LOM (eV)	Energy barrier of the PDS in AEM (eV)
Fe, F-CoO	1.17	1.22
F-CoO	1.52	1.53
Fe-CoO	2.50	1.38
CoO	2.72	2.01

Supplementary Table 8. Comparison of the overall water splitting performance of Fe, F-CoO NNAs-based system with the state-of-the-art electrolyzers.

Electrolyzers	Potanial$_{10}$ (V)	Potanial$_{100}$ (V)	Potanial$_{500}$ (V)	References
Fe, F-CoO NNAs|| Pt/C	1.41	1.57	1.70	This work
Pt/C/$Fe_{0.4}Ni_{0.6}$||$Fe_{0.4}Ni_{0.6}$	1.49	/	/	Appl. Catal. B: Environ. 2021, 286, 119902.
S-FeOOH||Pt/C	1.50	/	/	Adv. Funct. Mater. 2022, 2112674.
W-$NiS_{0.5}Se_{0.5}$||W-$NiS_{0.5}Se_{0.5}$	1.44	1.55		Adv. Mater. 2022, 34, 2107053.
V−CoP_2/CC||V−CoP_2/CC	1.47	/	/	Angew. Chem. Int. Ed. 2022, 61, e202116233.
$Ni_xFe_yCo_{6-x-y}Mo_6C$/NF|| $Ni_xFe_yCo_{6-x-y}Mo_6C$/NF	1.47	/	1.77	Appl. Catal. B: Environ. 2021, 290, 120049.
NFO-S5||NFO-S60	1.43	/	/	Appl. Catal. B: Environ. 2022, 305, 121030.
(Ni,Co)Se_2-GA||(Ni,Co)Se_2-GA	1.60	/	/	ACS Catal. 2017, 7, 6394.
LSC/K-$MoSe_2$||LSC/K-$MoSe_2$	1.59	1.95	/	Nat. Commun. 2021, 12, 4606.
CF/VMFO||CF/VMFO	1.37	1.59	/	Nat. Commun. 2021, 12, 1380.
NiCoP||NiCoP	1.65	1.82	/	Adv. Energy Mater. 2023, 2300499.
Fe-CoP/NF||Fe-CoP/NF	1.49	/	/	Adv. Sci. 2018, 5, 1800949.
Ni-MoN||SSM	1.61	1.71	/	Adv. Mater. 2022, 2201774.
NW-$MnCo_2O_4$/GDY||NW-$MnCo_2O_4$/GDY	1.47	1.60	/	Adv. Funct. Mater. 2021, 2107179.
Ni-Mo-B HF||Ni-Mo-B HF	1.39	1.62	1.88	Adv. Funct. Mater. 2021, 2107308.

4. Supplementary References

1 Perdew J. P. *et al.*, Generalized gradient approximation made simple, *Phys. Rev. Lett.* **77**, 3865-3868 (1996).

2 Monkhorst H. J. & Pack J. D., Special points for Brillouin-zone integrations, *Phys. Rev. B* **13**, 5188-5192 (1976).

3 Wang H. et al., Hydrogenated TiO_2 nanoparticles loaded with Au nanoclusters demonstrating largely enhanced performance for electrochemical reduction of nitrogen to ammonia, *Energy Technol.* **10**, 2200085 (2022).

4 Farsi L. & Deskins N. A., First principles analysis of surface dependent segregation in bimetallic alloys, *Phys. Chem. Chem. Phys.* **21**, 23626-23637 (2019).

5 Liu, M. et al. Enhanced electrocatalytic CO_2 reduction via field-induced reagent concentration. *Nature* **537**, 382 (2016).

6 Wang, J. D. & Hume, W. R. Diffusion of hydrogen ion and hydroxyl ion from various sources through dentine. *Int. Endod. J.* **21**, 17-26 (1988).

7 Friedman, A. M. & Kennedy, J. W., The self-diffusion coefficients of potassium, cesium, iodide and chloride ions in aqueous solutions, *J. Am. Chem. Soc.* **77**, 4499 (1955).

8 Li, J. F. et al. Surface analysis using shell-isolated nanoparticle-enhanced Raman spectroscopy. *Nat. Protoc.* **8**, 52-65 (2013).

9 Lee S. et al. Oxygen isotope labeling experiments reveal different reaction sites for the oxygen evolution reaction on nickel and nickel iron oxides. *Angew. Chem. Int. Ed.* **131**, 10401-10405 (2019).

10 Zhang N. et al. Lattice oxygen activation enabled by high-valence metal sites for enhanced water oxidation. *Nat. Commun.* **11**, 4066 (2020).
