# Supplementary material for: Lattice oxygen activation and local electric field enhancement by co-doping Fe and F in CoO nanoneedle arrays for industrial electrocatalytic water oxidation
Source: Nat Commun. 2024 Feb 3;15:1012. doi: 10.1038/s41467-024-45320-0 (PMC10837452; doi:10.1038/s41467-024-45320-0)
Supplement: Supplementary file 3 — Description of Additional Supplementary Files [file 41467_2024_45320_MOESM3_ESM.pdf]

<u>Description of Additional Supplementary Files</u>

Supplementary Movie Legends

Supplementary Movie 1: In situ optical microscope movie of Fe, F-CoO NNAs in 1 M KOH.

Supplementary Movie 2: In situ optical microscope movie of CoO NNAs in 1 M KOH.
